# Outcomes from the implementation of a counselling model supporting rapid antiretroviral treatment initiation in a primary healthcare clinic in Khayelitsha, South Africa

**DOI:** 10.4102/sajhivmed.v16i1.367

**Published:** 2015-07-02

**Authors:** Lynne Wilkinson, Helene Duvivier, Gabriela Patten, Suhair Solomon, Leticia Mdani, Shariefa Patel, Virginia de Azevedo, Saar Baert

**Affiliations:** 1 Médecins Sans Frontières, Khayelitsha Project, South Africa; 2 Médecins Sans Frontières, South African Mission, South Africa; 3 City of Cape Town Health Department, Khayelitsha, South Africa; 4 Médecins Sans Frontières, South African Medical Unit, Belgium

## Abstract

**Background:**

Lengthy antiretroviral treatment (ART) preparation contributes to high losses to care between communicating ART eligibility and initiating ART. To address this shortfall, Médecins Sans Frontières implemented a revised approach to ART initiation counselling preparation (integrated for TB co-infected patients), shifting the emphasis from pre-initiation sessions to addressing common barriers to adherence and strengthening post-initiation support in a primary healthcare facility in Khayelitsha, South Africa.

**Methods:**

An observational cohort study was conducted using routinely collected data for all ART-eligible patients attending their first counselling session between 23 July 2012 and 30 April 2013 to assess losses to care prior to and post ART initiation. Viral load completion and suppression rates of those retained on ART were also calculated.

**Results:**

Overall, 449 patients enrolled in the study, of whom 3.6% did not return to the facility to initiate ART. Of those who were initiated, 96.7% were retained at their first ART refill visit and 85.9% were retained 6 months post ART initiation. Of those retained, 80.2% had a viral load taken within 6 months of initiating ART, with 95.4% achieving viral load suppression.

**Conclusions:**

Adapting counselling to enable rapid ART initiation is feasible and has the potential to reduce losses to care prior to ART initiation without increasing short-term losses thereafter or compromising patient adherence.

## Introduction

One-third of antiretroviral treatment (ART)-eligible patients are estimated to be lost to care between communicating ART eligibility and initiating ART^1^ – the so-called ‘third stage’ of pre-ART care^2^ – increasing the risk of morbidity and mortality.^3^ Whilst patient education and adherence counselling are recommended to improve long-term adherence,^4,5^ lengthy preparation processes before starting ART cause delays during this pre-ART stage, contributing to high losses to care.^6,7^

In practice, there are wide-ranging approaches to patient ART preparation within the South African public sector.^6^ In 2012, the South African National Department of Health released a circular that recommended fast-tracking patients onto ART without unnecessary delay. It further recommended that patients with CD4 counts < 200 cells/mm^3^ and pregnant women be started on the same day that ART eligibility is ascertained. Some facilities now provide minimal ART preparation owing to prioritising fast-tracking, whilst others continue to require prior attendance of 3 (and sometimes more when co-infected with TB) education and adherence counselling sessions.

Médecins Sans Frontières (MSF), along with its partners (see acknowledgements), developed a revised approach to ART initiation counselling which supports rapid initiation without failing to adequately prepare a patient for lifelong adherence to treatment, including those who are co-infected with TB or pregnant. The overall aim of the revised approach is to reduce the loss of patients prior to ART initiation without increasing losses post ART initiation or reducing adherence amongst such patients.

The aim of the present study is to describe the intervention and determine the retention outcomes during the third stage of pre-ART care and post ART initiation of patients who underwent this model of counselling. Secondary outcomes include viral load completion and suppression rates of those retained on ART.

## Methods

### Study design

This was an observational cohort study using routinely collected data.

### Study setting

Khayelitsha is a peri-urban township on the outskirts of Cape Town, South Africa. It has a population of approximately 500 000 with high burdens of both HIV and TB. In 2011, the antenatal HIV prevalence was 34.3%^8^ and the TB case notification rate at least 1500 per 100 000 population per year.^9^

The study site (Kuyasa Clinic) is a general primary healthcare facility run by the City of Cape Town's Health Department. HIV and TB related services include: HIV counselling and testing (HCT), pre-ART, ART initiation, ART management, ART adherence clubs^10^ and both drug-susceptible and drug resistant TB services. According to 2013/2014 records, the clinic tests each month approximately 466 patients for HIV (2014), initiates approximately 50 patients on ART (2014), starts TB treatment for approximately 37 patients (2013) and had 2766 ART patients retained in care at the end of June 2014, with 772 (28%) receiving their treatment and care through ART adherence clubs.

### Antiretroviral treatment management

In South Africa, ART eligibility is determined by World Health Organization clinical staging combined with CD4 cell count. Western Cape HIV clinical guidelines provided that, from August 2011, patients with WHO stage 4 disease or those with a CD4 count < 350 cells/mm^3^ were eligible to start ART. Patients are due for their first and second viral loads at 4 and 12 months on ART respectively.

### Antiretroviral treatment preparation education and counselling

Prior to July 2012, the ART initiation counselling model in place at Kuyasa Clinic consisted of three counselling sessions, scheduled at weekly intervals after communication of ART eligibility, followed by ART initiation. Co-infected TB/HIV patients were also required to undergo 3 TB counselling sessions, which at the time were not integrated within the ART preparation counselling process. Where a clinician indicated a need to rapidly initiate HIV or TB treatment for clinical reasons, counselling was condensed into fewer sessions. Counsellors were not trained on how to appropriately condense sessions – specifically on what information remained pertinent and necessary. Counselling was provided by lay counsellors, employed by a nongovernmental organization (NGO) funded by the Western Cape's Department of Health (WCDoH). The counsellors were trained by the WCDoH's AIDS Training, Information and Counselling Centre (ATICC) according to Egan's ‘skilled helper’ model of counselling.^11^

**FIGURE 1:**
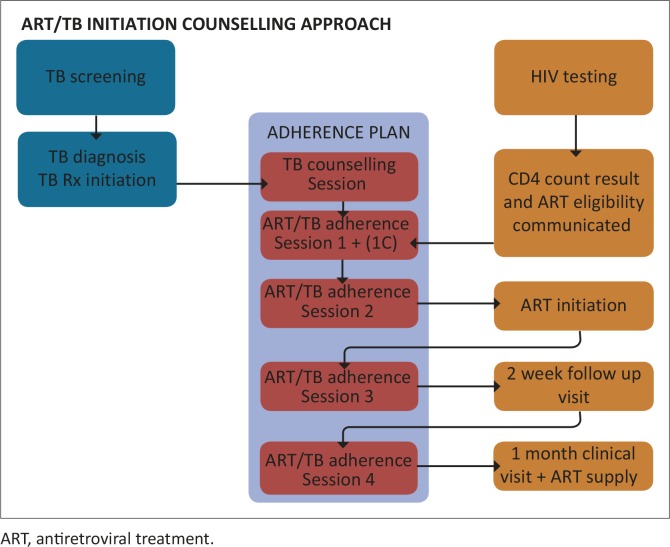
Timing of counselling sessions. ART, antiretroviral treatment

From July 2012, MSF supported the implementation of a revised ART initiation counselling model at Kuyasa Clinic. In summary, the lay counsellor provides a total of four counselling sessions: 1 session prior to ART initiation (‘session 1’) on the date when ART eligibility is communicated to the patient, 1 session on the day of ART initiation (‘session 2’), and two sessions post ART initiation ('sessions 3 and 4’) on subsequent clinic appointment/ART refill dates. Where the patient indicates at the end of session 1 that he/she is not ready to start, a further ‘not ready to start’ session is scheduled ('session 1C’) that specifically aims to work through patient-identified barriers to initiating ART. Where it is appropriate for clinical reasons to initiate ART on the day that ART eligibility is assessed, it is possible to carry out the first two sessions on the same day. Where a patient is diagnosed with TB and HIV, one additional counselling session is added prior to session 1 to prepare the patient for starting and adhering to TB treatment. Each counselling session is designed to take 12 min – 18 min. Figure 1 outlines session timing. Where a patient is not able to return to the clinic 2 weeks after ART initiation, sessions 3 and 4 are combined and provided at the first ART refill date, which is 28 days after ART initiation.

The counselling approach is based on the Life Steps intervention to HIV medication adherence^12,13^ in which cognitive behavioural, problem solving, and motivational interviewing techniques are used to enhance motivation and assist HIV-positive patients to develop better skills for adhering to HIV treatment. This model has shown favourable results in different settings.^14^

The Life Steps model was simplified and adapted to barriers and adherence planning requirements identified by MSF through its work with patients attending a second-line failure clinic in Khayelitsha.^15,16^ Patient education is limited to essential information to initiate ART and TB treatment, supported through the use of a visual aid and a take-home leaflet. The 14 most common barriers to start ART and adherence (Box 1) are addressed with all patients and are documented in each patient's adherence plan. The approach focuses on creating a patient-oriented motivation for commitment to lifelong treatment.

**BOX 1:** Adherence steps addressed in antiretroviral treatment initiation counselling model.

14 adherence steps:	
Step 1:	Understanding HIV (and TB)
Step 2:	Identify support system
Step 3:	Planning future appointments
Step 4:	Readiness to start treatment
Step 5:	Creation of a medication schedule
Step 6:	Managing missed doses
Step 7:	Reminder strategies
Step 8:	Storing medication and extra doses
Step 9:	Dealing with side-effects
Step 10:	Planning trips
Step 11:	Dealing with substance use
Step 12:	Communication with treatment team
Step 13:	Learning from mistakes
Step 14:	Making goals: suppressed viral load (and TB continuation phase)

Session guides were developed, defining the content to address per session, and served as a working tool for both counsellor and counsellor supervisor. Both counsellors were trained, and fidelity to the intervention was assured through regular supervision by observation of sessions using a standardised observation checklist, and review of patients’ adherence plans and counsellors’ paper registers recording patients enrolled and counselling sessions provided. This supervision was performed by an NGO counsellor supervisor who covered a number of clinics and MSF staff.

For further details on the model, including session plans and tools, see ART/TB/PMTCT Initiation counselling model report and toolkit at http://bit.ly/197VDfb.

### Study participants

From 23 July 2012 to 30 April 2013, all HIV-positive patients found to be eligible for ART initiation who attended session 1were enrolled in the study.

### Data collection and analysis

All data were collected (other than viral load results) at the end of January 2014, providing a minimum of 9 months of follow-up. Viral load data were collected on 30 September 2014. Variables collected included age, sex, CD4 count, TB at ART start, TB treatment start date, attendance of counselling sessions, ART initiation date, last visit date to the clinic for ART refill, reported transfers and deaths, and dates of first viral load and first viral load outcome.

Study outcomes were time taken and retention from session 1 to ART initiation, short-term retention on ART at 28 days (first ART refill) and 6 months (183 days) post ART initiation, viral load completion and suppression within 6 months of ART initiation, and viral load suppression at first viral load.

We defined retention at 1 month and 6 months as those patients whose last visit to the clinic or documented transfer date was more than 28 days and 6 months after the date of ART initiation. Viral load suppression was defined as < 400 copies/mL.

Descriptive analyses were done in STATA Version 13.^17^

### Ethics approval

Ethics approval was obtained from the University of Cape Town Human Research Ethics Committee. Permission to do the study was obtained from the City of Cape Town research committee.

## Results

A total of 449 patients attended session 1 from 23 July 2012 to 30 April 2013, thereby enrolling in the study, of whom 300 (66.8%) were female and 137 (30.5%) of whom had TB at enrolment. Median age was 31 years (interquartile range [IQR] 26–37 years) and median CD4 count 242 cells/mm^3^ (IQR 147 cells/mm^3^ – 308 cells/mm^3^) at enrolment. A further breakdown of age and CD4 count categories is set out in Table 1.

**TABLE 1:** Demographic and clinical characteristics of patients included in study at enrolment.

Characteristics	Number	%
**Gender**	
Male	149	33.2
Female	300	66.8
**Age (in years)**	
Median†	31	-
14–18	19	4.2
19–25	80	17.8
26–39	272	60.6
40–54	75	16.7
> 55	3	0.7
**CD4 count‡ (cell/mm^3^)**	
Median§	242	-
≤ 50	33	7.4
51–200	131	29.6
201–350	224	50.6
351–500	36	8.1
> 500	19	4.3

*N* = 449.

†, IQR 26–37; ‡, missing three patients’ CD4 counts; §, IQR 147–308.

**FIGURE 2:**
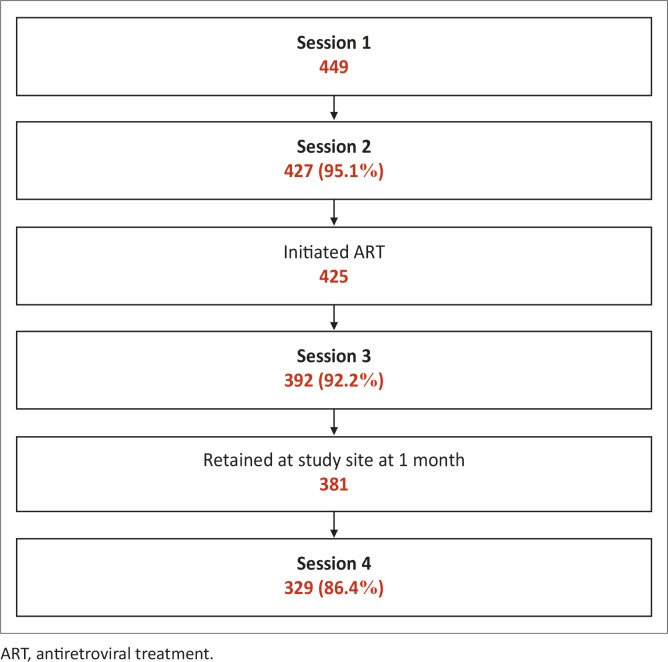
Counselling session completion. ART, antiretroviral treatment.

Figure 2 presents a summary of counselling session completion. Of those patients who enrolled in the study, 427 (95.1%) completed session 2, of whom two did not proceed to initiate ART. Session 3 was completed by 392 (92.2%) of those who completed session 2 and initiated ART and 329 (86.4%) of those patients who returned to the study site for their 28-day ART refill, completed session 4.

**FIGURE 3:**
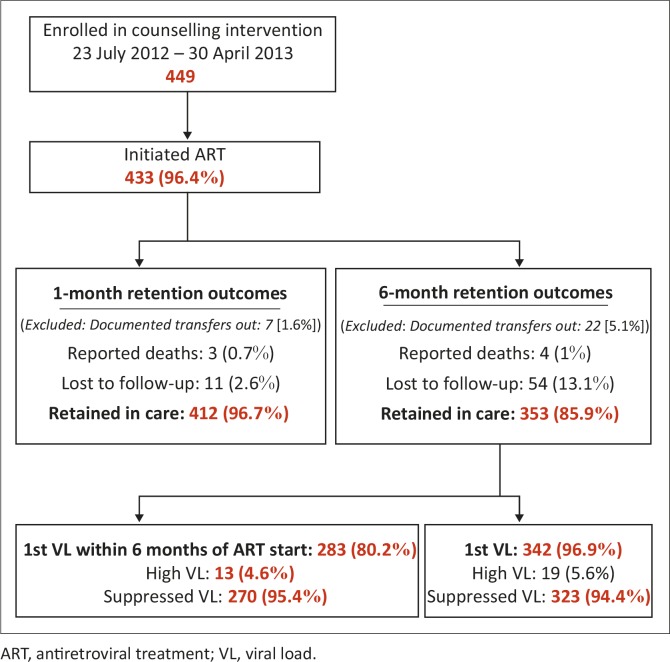
Retention and viral load outcomes. ART, antiretroviral treatment; VL, viral load.

Figure 3 details retention in care from enrolment to 6 months post ART initiation. ART was initiated by 433 (96.4%) patients. Median time to the start of ART for all enrolled patients from session 1 was 5 days (IQR 2–14 days). Forty-nine (11%) patients started ART on the same day as session 1, indicating fast-tracking for clinical reasons including pregnancy, whilst 32 (7%) took more than 28 days to start (see further breakdown of time to ART start in Table 2 below). Patients with a concurrent TB diagnosis took a median of 18 days (IQR 14–29 days) from start of TB treatment to initiate ART. After excluding patients with documented transfers to other facilities, 412 (96.7%) and 353 (85.9%) patients were retained respective at 1 and 6 months post ART initiation.

**TABLE 2:** Time from enrolment (session 1) to antiretroviral treatment start and first viral load.

Category	Subcategory	Number	Percentage
Enrolment (session 1) to antiretroviral treatment start†	Median (IQR)	5 days (IQR 2–14 days)	-
	Same day	49	11
	1–7 days	224	52
	8–14 days	55	13
	15–21 days	47	11
	22–28 days	16	4
	> 28 days	32	7
	Counselled after antiretroviral treatment start	5	1
	Restarted (treatment interrupters from other antiretroviral treatment sites)	5	1
Time from antiretroviral treatment start to first viral load‡	Median days (IQR)	134.5 (114–174)	-
	≤ 6 months	283	80.2
	6–9 months	33	9.3
	9–≤ 13 months	19	6.7
	> 13 months	7	2.0
	No viral load taken by 30 September 2014	11	3.1

†, *N* = 433; ‡, *N* = 353.

The median time to first viral load was 4.4 months (134.5 days [IQR 114–174 days]). Of those patients retained at the study clinic, 283 (80.2%) had a viral load taken within 6 months of initiating ART, with 270 (95.4%) achieving viral load suppression. By 30 September 2014, 342 (96.9%) had their first viral load taken, with a 94.4% suppression rate. See Table 2 for a further breakdown of time to first viral load.

## Discussion

By adapting ART initiation education and counselling to expedite the start of treatment, whilst addressing common barriers to patients’ readiness to start ART, only 3.6% of patients were lost from care between their first counselling session and ART initiation. The proportion of patients initiating ART after being informed of their eligibility for treatment is substantially higher than estimates reported previously in South Africa and elsewhere in the region.^1,18^ Our findings are comparable to the outcomes of a randomised controlled trial by Rosen et al. which reported pre-ART losses when rapid (2%) versus standard ART initiation (28%) took place.^19^

Reducing losses to care prior to ART initiation carries a potential risk of increased losses immediately after ART start, if patients are uncomfortable with starting quickly or are insufficiently prepared. Our study found limited losses immediately after ART start, with 2.6% of those initiated not returning for their 1-month ART refill appointment. At 6 months after ART initiation, 85.9% of patients were retained, which is comparable to elsewhere in South Africa^20^ and sub-Saharan Africa.^21,22^ Whilst cohorts that started ART prior to 2008 in Khayelitsha were reported to have higher 6-month retention outcomes,^23^ a recent study reporting on MSF cohorts has shown higher losses to care in more recently initiated cohorts, with the 2011 cohort retaining 85.5% (95% CI 84.5% – 86.4%) at this time point.^22^ It is worth noting that it may not be appropriate to compare retention reported in older versus more recently initiated cohorts; this point is due to increasing unaccounted-for self-transfers hidden within the lost to follow-up (LTFU) outcome^24^ and a bias towards better retention outcomes in older cohorts introduced by longer follow-up times, which allow transient treatment interrupters to return to care.^25^

It took less than a week for patients to initiate ART from starting ART preparation counselling. Whilst there is extensive evidence on the delay between ascertaining ART eligibility (date of CD4 count) and initiating ART, with a recent systematic review reporting a range of 22–108 days,^18^ we could find no routine programme evidence specifically measuring the time from starting ART preparation counselling to ART initiation.

High rates of both viral load completion and suppression were achieved. These were higher than those previously reported for ART patients in Khayelitsha.^23^ Focusing the ART initiation counselling sessions on making practical plans to overcome the most commonly experienced barriers to maintaining good treatment adherence, together with educating and motivating patients towards the goal of achieving an undetectable viral load, might have contributed to these high completion and suppression rates.

The use of motivational interviewing has been recommended to ensure behaviour change related to ART adherence^26^ but, in resource-limited settings, major challenges exist to make lay counsellors proficient in using these more complex counselling techniques.^27,28^ Whilst this model was based on principles of motivational interviewing, by engaging with all patients on planning around the same common barriers to adherence, the counselling model did not require lay counsellors to use more complex counselling techniques. Barriers to address with patients were predefined, and each adherence barrier was addressed in a standardised way by setting the goal, identifying the possible barriers to reaching that goal, and concluding on a plan per adherence barrier. Our experiences with the ART initiation counselling model suggest that this may provide a feasible approach to enhancing the counselling skills of lay counsellors, whilst taking their limitations into account. The present study is not comparable with a recently published study in South Africa that found no difference in patient adherence or virological suppression outcomes when assessing those who received only didactic education with those who received both didactic education and counselling utilising motivational interviewing skills.^29^ Our study was carried out in an operational setting, provided a maximum of one session prior to the day of ART initiation, sessions were considerably shorter in length, and sessions were carried out by existing lay counsellors only, and not nursing staff.

Our results should be considered in the light of the following strengths and limitations: the principle strength of the study is demonstration of feasibility within an operational setting utilising existing lay counselling staff. There were a number of limitations, the first three relating to the study constraint of limited pre-ART routine data. Firstly, we were unable to provide a comparison group as a result of poor routine pre-ART data collection prior to implementation of the revised ART initiation counselling model, including attendance of ART counselling preparation sessions. Secondly, the date of baseline CD4 counts was not accurately captured, often reflected as the same date as ART initiation, limiting our capacity to calculate length of time from eligible CD4 count to ART start. Thirdly, whilst the intervention was designed for patients to attend session 1 immediately after being informed of their ART eligibility, we were unable to verify the date on which such communication took place. Further limitations should be noted: during the evaluation period described by the present study, the counselling model had not yet been specifically adapted for pregnant women initiated on ART. Whilst these women were enrolled in the study and accounted for some of the same-day ART initiations with high CD4 counts, we were unable to reliably identify those starting ART for PMTCT purposes. Their inclusion in the study cohort might have increased overall short-term losses to care as reported elsewhere.^30,31^ Lastly, despite basic interventions to ensure fidelity, no specific data were collected to report on fidelity to the piloted model.

The present study highlights useful directions for future research. Most importantly, further evidence is required to determine the optimal ART initiation counselling model that can feasibly be implemented in settings with lay counsellors, who have no formal professional or paraprofessional degree in counselling and high numbers of ART patients. These models should ideally also evaluate pre- and post ART retention outcomes. To provide a better understanding of the counselling model, components such as timing, counselling provider, session length, content and measures to ensure fidelity to the intervention should always be described. In addition to counselling model research, more studies are required that report on the extent of delays and losses to care attributable to lengthy ART preparation processes prior to ART initiation. Lastly, whilst there is substantial evidence on optimal timing for starting ART after TB treatment initiation from a clinical perspective,^32,33^ competing risk analysis would benefit from studies reporting on losses to care caused by delaying ART initiation for TB co-infected patients.

## Conclusion

Adapting initiation education and counselling to enable the rapid start of ART, by addressing common barriers to adherence and strengthening post-initiation support, is feasible. It has the potential to reduce losses to care prior to ART initiation without increasing short-term losses thereafter or compromising patient adherence. ART programmes should consider adjusting their ART initiation counselling to limit delays but ensure that fast-tracking does not result in patients receiving inadequate adherence support with possible negative long-term consequences.
